# Molecular Phylogeny of the Genus *Lolliguncula* Steenstrup, 1881 Based on Nuclear and Mitochondrial DNA Sequences Indicates Genetic Isolation of Populations from North and South Atlantic, and the Possible Presence of Further Cryptic Species

**DOI:** 10.1371/journal.pone.0088693

**Published:** 2014-02-25

**Authors:** João Bráullio L. Sales, Unai Markaida, Paul W. Shaw, Manuel Haimovici, Jonathan S. Ready, Wilsea M. B. Figueredo-Ready, Fabricio Angioletti, Manoela A. Carneiro, Horacio Schneider, Iracilda Sampaio

**Affiliations:** 1 Universidade Federal do Pará, Campus Universitário de Bragança, Bragança, Pará, Brazil; 2 Línea de Pesquerías Artesanales, El Colegio da la Frontera Sur, Lerma, Campeche, Mexico; 3 Institute of Biological, Environmental and Rural Sciences, Aberystwyth University, Aberystwyth, Dyfed, United Kingdom; 4 Laboratório de Recursos Demersais e Cefalópodes, Universidade Federal do Rio Grande, Caixa Postal 474, Rio Grande, Rio Grande do Sul, Brazil; Institut Pasteur, France

## Abstract

Squid of the genus *Lolliguncula* Steenstrup, 1881 are small bodied, coastal species capable of tolerating low salinity. *Lolliguncula* sp. are found exclusively in the New World, although only one of the four recognized species (*Lolliguncula brevis*) occurs in the Atlantic Ocean. Preliminary morphological analyses suggest that *Lolliguncula brevis* populations in the North and South Atlantic may represent distinct species. The principal objective of the present study was to verify the phylogenetic relationships within the genus and test for the presence of possible cryptic species. Both gene and species tree topologies indicated that *Lolliguncula brevis* specimens from the North and South Atlantic represent distinct phylogenetic clades. In contrast with previous studies, *L. panamensis* was identified as the basal species of the genus. Our results provide important insights into the phylogenetic relationships among the *Lolliguncula* specimens analyzed, and confirm the genetic separation of *Lolliguncula brevis* populations of the North and South Atlantic at the level of sister species.

## Introduction

The genus *Lolliguncula*, Steenstrup, 1881 was derived from the separation of *Lolliguncula brevis*, Blainville, 1823 from the genus *Loligo* [1]. The squids of the genus *Lolliguncula* are small in size and occur in warm, shallow coastal waters, and are the only cephalopods found in brackish water [2]. In the 1980s and 1990s, a number of taxonomic advances were made, including the description of a number of new species [3], [4], and the reclassification of *Lolliguncula mercatoris* Adam, 1941, the only member of the genus not distributed in the western hemisphere, to a new genus *Afrololigo* Brackoniecki, 1986. *Afrololigo* was initially defined on the basis of the morphology of the hectocotylus, although the subsequent analysis of DNA sequences [5] validated the separation of the two genera.

Four *Lolliguncula* species are recognized at the present time, representing two subgenera found exclusively in the western hemisphere – *Lolliguncula* (*Lolliguncula*) *brevis*, Blainville, 1823; *Lolliguncula* (*Lolliguncula*) *argus*, Brakoniecki & Roper, 1985; *Lolliguncula* (*Lolliguncula*) *panamensis* Berry, 1911, and *Lolliguncula* (*Loliolopsis*) *diomedeae* Hoyle, 1904 [6]. Of these species, only *Lolliguncula brevis* is found in the Atlantic Ocean, ranging between Nova Scotia, Canada, in the north Atlantic and southern Brazil in the south Atlantic. This species is found in relatively shallow waters, of less than 20 m in depth, and is tolerant of low salinity [2]. Studies conducted in the 1990s [7] indicated differences in the morphology and spawning patterns of *Lolliguncula brevis* populations in the northern and southern hemispheres, raising doubts with regard to the taxonomic validity of *Lolliguncula brevis*, in particular that specimens from the northern and southern Atlantic may represent distinct species [8].

Anderson [5] was the first to provide phylogenetic inferences on the members of the genus *Lolliguncula*, although this analysis did not include specimens from South America and was based solely on mitochondrial genes. The present study aimed to test whether *Lolliguncula brevis* is a single species throughout its geographic distribution and provides a molecular phylogenetic framework for systematic analyses of the genus.

## Methods

### Specimen Collection and the Genes Analyzed

The present study analyzed three of the four recognized *Lolliguncula* species. Specimens of *Lolliguncula panamensis* were collected from the northeastern Pacific and *Lolliguncula brevis* from the South Atlantic. DNA sequences generated from these specimens were analyzed together with sequences available in GenBank for other species of loliginid squid [9], [10], [11], [12] (Table S1). All samples in this study were collected following national regulations and laws for collection of scientific specimens in each country. No ethics committee approval is required for these organisms in Brazil and Mexico at this time. Capture, transport and handling of the specimens in Brazil was conducted under ICMBio license N° 20261-1. No special permits were obtained for samples collected in Mexico (*L. panamensis*) since the Mexican government does not require licences or authorization for capture, handling or scientific sampling of Cephalopoda, given that these species are not at risk of overfishing. They are not a target of commercial fisheries (Unai Markaida, pers. comm.). However, to receive these samples in Brazil an import licence for biological material was required. This was obtained through IBAMA (Brazilian Institute for the Environment and Natural Resources - license N° 12BR009032/DF). Specimens were handled following literature suggestions summarized in a review for aquaculture of cephalopods [13] and in a recent work with Cephalopods [5], [8]. Samples were obtained (already deceased) either from fishermen or, when collected alive they were anaesthetized and euthanased immediately following the 3R’s (replacement, reduction and refinement) concept and existing knowledge for cephalopods. Gradual addition of cooler water lowers body temperature, anaesthetizing the animals [13]. When activity ceases, animals are frozen as a second step to complete euthanasia (S6.3, page 74, AVMA Guidelines for the Euthanasia of Animals: 2013 Edition). In Brazil any ethical considerations for capture, transport and handling of scientific samples is linked directly into the application for licenses issued by IBAMA and ICMBio, which were obtained as previously indicated. Although there are various types of ethics committees in universities and other institutions, they only refer to experiments with live animals, for which distinct laws do exist see http://www.mct.gov.br/upd_blob/0226/226746.pdf).

The specimens collected in the Brazilian state of Pará were captured during the day, approximately 50 m from the coast, during the spotted pink shrimp (*Penaeus brasiliensis*) harvest. The specimens from the Brazilian state of Paraíba were captured with a bottom drag-net during the early morning using lanterns focused directly into the water along the edge of the beach. The samples from the Brazilian state of Bahia were collected using alternative techniques at the different localities, with the specimens from Jequié and Praia de Guaiabin being collected close to the beach in drag-nets at low tide, while those from Caravelas were obtained from bottom-trawling fishing boats.

Tissue samples were stored in ethanol prior to the extraction of the DNA, and voucher specimens were preserved in 10% formalin for morphological analyses. Total genomic DNA was extracted with the Wizard Genomic DNA Purification Kit (Madison, WI), followed by the extraction protocol for animal tissue (mouse tail). Each sample was washed twice in 600 µl of sterile ultra-pure double distilled water by refrigerated centrifugation at 16,000 rpm during 2 minutes (Sigma Aldrich, 2K15). The mitochondrial 16S rDNA and Cytochrome Oxidase subunit I (COI) genes and the nuclear Rhodopsin gene were chosen due primarily to the fact that these markers have proven to be adequate for phylogenetic reconstruction in other loliginid species [9]. The PCRs were run in a final volume of 25 µl containing a mixture of 0.5 µl of each primer, 2 µl of MgCl2 (25 mM), 4 µl of the dNTP mixture (1.25 mM), 5.0 µl of 5× buffer (Promega, Madison-WI USA-Tris-HCl and KCl, pH 8.5), 0.2 µl of Taq polymerase (5 U/µl, Promega, Madison-WI USA), approximately 100 ng of the total DNA, with ultra-pure water to complete the final volume. The amplification of the mitochondrial 16S gene was based on the following cycling parameters: 2 minutes at 94°C for denaturation, followed by 30 cycles of 30 seconds at 94°C, 1 minute at 51°C for annealing, 2 minutes at 72°C for extension, and then 7 minutes at 72°C for final extension. For COI, the cycle was 2 minutes at 94°C for denaturation, followed by 30 cycles of 1 minute at 94°C, 1 minute at 45.5°C for annealing, 2 minutes at 72°C for extension, and then 7 minutes at 72°C for final extension. For the rhodopsin gene, the parameters were 15 minutes at 95°C for denaturation, followed by 35 cycles of 1 minute at 94°C, 1 minute at 61°C for annealing, 1 minute and 30 seconds at 68°C for extension, and 7 minutes at 72°C for final extension. For sequencing, the samples were purified with the ExoSAP-IT enzyme (Amersham Pharmacia Biotech Inc.). The sequencing reactions were conducted using reagents from the BigDye kit (Applied Biosystems), and the samples were then sequenced in an ABI 3500 automatic sequencer (Applied Biosystems).

### Sequence Alignment and Phylogenetic Reconstruction

All sequences were initially aligned using CLUSTALW [14] in BioEdit version 5.0.6 [15]. The alignments were subsequently checked visually to correct any possible errors. Maximum likelihood and Bayesian analyses were conducted using separated or concatenated datasets. For Bayesian analyses the datasets were partitioned by gene (16S, COI and Rhod) and by codon (COI and Rhod).

Maximum likelihood analyses were run for each dataset individually with an evolutionary model selected by each gene and with the three genes concatenated with one evolutionary model chosen for the whole dataset. As an alternative for the concatenation of the three genes a multi-locus coalescent analysis for inference of species trees from multilocus data was employed using *BEAST (Star BEAST) [16]. Bayesian Inference (BI) and Maximum likelihood analyses were run using MrBayes 3.1.2 [17], and PhyML v3.0 [18], respectively. jModelTest [19] was used to select the best evolutionary model for each dataset. Genetic distances were calculated in PAUP* [20] using uncorrected (“p”) distances.

The statistical reliability of the arrangements was attained by bootstrap analysis (using 1000 replicates) [21] and posterior probability in likelihood and Bayesian analyses, respectively. In MrBayes 3.1.2 [14], analyses were based on the Markov chain Monte Carlo (MCMC) sampling procedure, with four simultaneous runs, each consisting of four chains (one cold, three heated), and a total run length of 10 million generations, using the parameters of the evolutionary models selected for each partition. The posteriori Bayesian probabilities were selected by the 60% consensus rule, with random starting trees and trees sampled every 5000 generations following the removal of the trees that appeared to have reached a stationary state, in which the burn-in was verified by the empirical examination of the likelihood values.

For species trees the same parameters were used employing different tools of the BEAST package [22] such as BEAUti for building the input file for BEAST; BEAST (in Star BEAST mode) for generating the a posteriori distribution of sampled trees; Tracer to evaluate continuous parameter values sampled from the chain; and TreeAnnotator for “summarizing the information from the sample of trees produced by BEAST onto a single “target” tree”.

## Results

### Concatenate and Multi Species Tree Analysis

**Figure 1:**
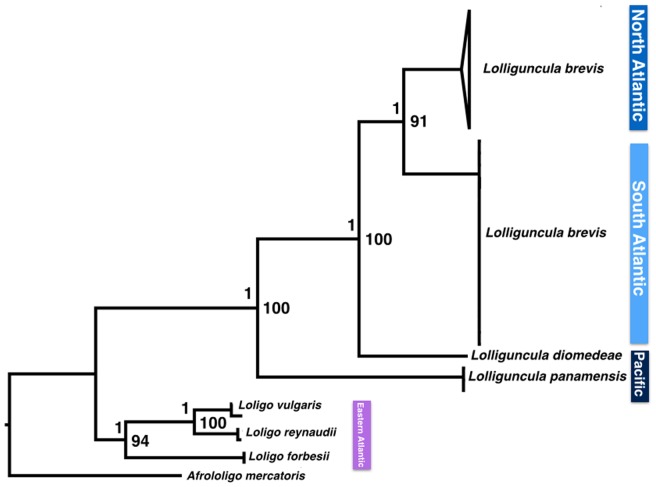
Maximum Likelihood Phylogenetic tree of the genus *Lolliguncula*. Topology based on the concatened datasets (16S+COI+Rhod). Bayesian credibility support and Bootstrap values are shown above and in front of the nodes, respectively.

The evolutionary models selected for each analysis are showed in Table 1. In both ML and Bayesian reconstructions the phylogenetic separation of *Lolliguncula brevis* specimens from the North and South Atlantic is strongly supported, with scores of 91% (ML) and 1 (BI) with *Afrololigo mercatoris,* and three species of *Loligo* genus (*L. vulgaris, L. reynaudii, L. forbesi*) used as outgroup (Fig. 1). *Lolligincula panamensis* appears as the most basal species of the genus, followed by *L. diomedeae*, which seems to be the sister group of the North and South Atlantic clade of *Lolliguncula brevis*. However, as was not possible to obtain samples from *Lolliguncula argus*, the fourth species of the genus, these assertions should be considered in the context of the present work.

**Table 1:** Summary of genetic markers and parameters and total sample number used in analyses.

Marker(s)	Length(base pairs)	Best-fittingmodel (AIC)	Best-fittingmodel (BIC)	Samplesincluded	PrimerReference/Primer sequence
16S	496 bp	TIM1+G	TIM1+G	24	[13]
COI	600 bp	TIM2+I	TIM2+I	24	[14]
Rhodopsin	646 bp	TIM2ef+G	TrNef	22	a/b
16S+COI+Rhod	1742 bp	GTR+G	TIM2+G	24	–

a) 5′- ARAAAATGAGCCACAGAAAG-3′; b) Rev 5′- TTSTTGYTGAGCCTGCATCTT-3′.

**Figure 2:**
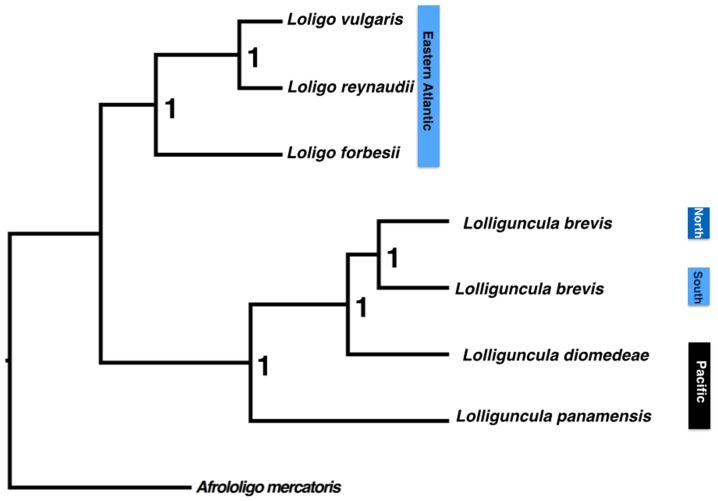
Partioned Bayesian Inference Phylogenetic tree of the genus *Lolliguncula*. Topology based on the concatened datasets (16S+COI+Rhod).

In the concatenated or species tree analysis practically no subdivisions inside the clades were observed (Figure 2). The only suggestion is the grouping of LbrSA668 and LbrSA672 with no significant support in both likelihood and Bayesian analysis (63% and 0.94, respectively: not showed) in spite of grouping significantly (ML = 92%; BI = 1) when just mtDNA genes were considered. But, as they are just two individuals of the same locality they do not provide any information on the genetic structure of the South Atlantic population.

### Nucleotide Divergences

**Figure 3:**
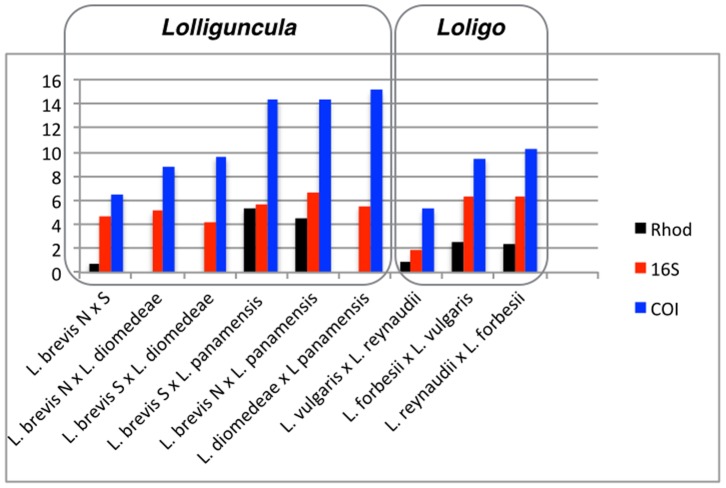
Multilocus species tree of the genus *Lolliguncula* based in two mitochondrial (16S and COI) and one nuclear genes (Rhod) obtained in the *BEAST program. Posterior probability values are shown at the nodes.

Non-corrected nucleotide divergences (P) were estimated for each dataset (See Table 2 and Fig. 3). At the intra specific level the average divergence estimated for the *Lolliguncula brevis* from the North Atlantic for the mtDNA and nucDNA genes were the following: 16S = 0.3% (min = 0; max = 1.1%), COI = 0.1% (min = 0 max = 0.3%); Rhod = no variation. For *Lolliguncula brevis* from the South Atlantic the divergences and range were: 16S = 0.1% (min = 0, max = 0.4%); COI = 0.1% (min = 0, max = 0.3%), and Rhod = no variation (Fig. 3).

**Table 2:** Genetic divergences (P) estimated for two mitochondrial and one nuclear genes in the present work.

	16S	2	3	4	5	6	7	8
1	*Afrololigo mercatoris*							
2	*Loligo forbesii*	0.066						
3	*Loligo reynaudii*	0.068	0.063					
4	*Loligo vulgaris*	0.069	0.063	0.018				
5	*Lolliguncula diomedeae*	0.058	0.083	0.072	0.071			
6	*Lolliguncula panamensis*	0.068	0.074	0.07	0.065	0.055		
7	*Lolliguncula brevis*	0.077	0.099	0.077	0.074	0.051	0.066	
8	*Lollicungula brevis*	0.066	0.096	0.082	0.074	0.042	0.057	0.046
	**COI**	**2**	**3**	**4**	**5**	**6**	**7**	**8**
1	*Afrololigo mercatoris*							
2	*Loligo forbesii*	0.13						
3	*Loligo reynaudii*	0.128	0.103					
4	*Loligo vulgaris*	0.128	0.095	0.053				
5	*Lolliguncula diomedeae*	0.162	0.149	0.154	0.157			
6	*Lolliguncula panamensis*	0.189	0.165	0.155	0.158	0.151		
7	*Lolliguncula brevis*	0.162	0.167	0.151	0.155	0.087	0.143	
8	*Lolliguncula brevis*	0.157	0.149	0.144	0.15	0.096	0.144	0.065
	**Rhod**	**2**	**3**	**4**	**5**	**6**	**7**	**8**
1	*Afrololigo mercatoris*	–						
2	*Loligo forbesii*	0.03	–					
3	*Loligo reynaudii*	0.03	0.023					
4	*Loligo vulgaris*	0.036	0.026	0.009				
5								
6	*Lolliguncula panamensis*	0.06	0.06	0.062		0.059	–	
7	*Lolliguncula brevis*	0.068	0.064	0.062		0.062	0.045	–
8	*Lolliguncula brevis*	0.073	0.068	0.064		0.064	0.053	0.008

7 = South Atlantic *Lolliguncula brevis*; 8 North Atlantic *Lolliguncula brevis*.

In contrast, the average nucleotide divergence between the populations of *Lolliguncula brevis* from North and South Atlantic, which were presumed to belong to a single species, were the following: 16S = 4.6% (min = 4.3%, max = 4.9%); COI = 6.5% (min = 6.3%, max = 6.7%); and Rhod = 0.8% (no intraspecific variation). These divergence values for the 16S gene are, in some cases, larger than, or in general similar to, those observed between congeneric species in the genera *Lolliguncula* and *Loligo*. For example, the divergence between *Loligo vulgaris* vs. *L. reynaudii* is 1.8%. (See Fig. 3, Table 2). Furthermore, the divergence between *L. diomedeae* vs. populations of *Lolliguncula brevis* from both the South and North Atlantic are 4.2% and 5.1%, respectively. Similarly, the divergence between *L. diomedeae* and *L. panamensis* is 5.5%, and the divergence between South Atlantic *Lolliguncula brevis* vs. *L. panamensis* is 5.7%. These values are approximately the same as that obtained in comparisons between *L. forbesii* and *L. vulgaris* or *L. reynaudii* (∼6.3%) (Table 2; Fig. 3).

In regard to the COI gene, the average divergence between *Lolliguncula brevis* populations from the North and South Atlantic was 6.5% (min = 6.3%, max = 6.7%). But, when these populations were compared to *L. diomedeae* the divergence varied from 8.7% to 9.6%, while the divergence between *L. diomedeae* and *L. panamensis* was 15.1%. On the other hand, the divergence between *L. reynaudii* and *L. vulgaris* was much lower (5.7%) and the divergence between *L. forbesii* and *L. reynaudii* was 9.5% and divergence between *L. forbesii* and *L. vulgaris* was 10.3% (Table 2; Fig. 3).

In relation to the nuclear gene Rhodopsin, the divergence between populations of *Lolliguncula brevis* from the North and South Atlantic was 0.8%, similar to the value observed between *Loligo reynaudii* and *L. vulgaris* (0.9%). But, the divergence values observed in other comparisons between *Loligo* species, *L. forbesii*×*L. reynaudii* (2.3%) and *L. forbesii*×*L. vulgaris* (2.6%) (Table 2; Fig. 3) were higher.

## Discussion

The results of the present study (the first molecular phylogenetic analysis to focus specifically on the genus) provide important insights into the phylogenetic relationships among the different members of the genus *Lolliguncula*. It was possible to establish the phylogenetic position of each species within the genus (except for *Lolliguncula* (*Lolliguncula*) *argus*, for which no sequences exist, nor are tissues available), providing an alternative interpretation of their relationships based on molecular data instead of morphology [1], [6].

The first key finding is that the data provides a new taxonomic viewpoint, with a basal position of *L. panamensis* (Figs. 1 and 2) rather than *L. diomedeae* (previously considered to be a distinct subgenus). The two species can be distinguished by the morphology of the males, and the shape of the fins in the females, as well as the fact that the two species are rarely captured together despite having a sympatric distribution [6]. Taxonomic uncertainties in relation to the subgenera *Lolliguncula* and *Loliolopsis* had been raised in previous studies due to their many shared morphological traits [4], [8], particularly similarities in the hectocotylus [4]. The results of the present study indicate that *L. diomedeae* lies within the *Lolliguncula brevis* - *L. panamensis* clade, making the *Loliolopsis* subgenus invalid.

The second key finding is the presence of a cryptic species within the geographic distribution of *Lolliguncula brevis*, in support of previous morphological studies [7]. All phylogenetic reconstructions indicated the genetic separation of *Lolliguncula brevis* from the North and South Atlantic, with significant statistical support and a sequence divergence of 4.6 and 6.5% for mtDNA (16S, COI) and 0.8% for nucDNA (rhodopsin), indicating that the specimens from the two hemispheres may represent sister species. Simone [7] identified variation in morphological features of *Lolliguncula brevis* specimens from the two hemispheres: southern specimens have smaller size, thinner mantle, a cylindrical mantle rounded towards the posterior extremity but extending no further than the base of the fins, and white or pale red coloration compared to a dark brown-reddish or yellow-chestnut coloration in the northern specimens [6], fewer suckers on the hectocotylus [4], and the presence of suckers on the buccal membrane. The distinct genetic differentiation of *Lolliguncula brevis* from the northern and southern Atlantic indicates a breakdown of gene flow, suggesting the presence of a physical barrier to dispersal between the hemispheres as observed in other loliginids [12]. In loliginid squid, genetic connectivity among populations may be influenced directly by either dispersal capacity of the planktonic larvae and/or mobility of the adults [6]. In comparison with other loliginids, *Lolliguncula brevis* has relatively large eggs and paralarvae, possibly indicating reduced dispersal [8]. Breakdown of widespread gene flow in *Lolliguncula brevis* may also be indicated from the identification of a number of local morphotypes by Zaleski [8], which may be related to reduced dispersal between inlets and estuaries in which populations of the species may become reproductively isolated along the southwest Atlantic coastline.

## Conclusions

The gene and species trees topologies generated in the present study support the classification of *L. panamensis*, rather than *L. diomedeae*, as the most basal species of the genus *Lolliguncula*, in context of the present work, contrasting, however with the findings of previous morphological studies [1]. The phylogenetic analyses of two mtDNA genes and one nucDNA gene also supported genetic separation of *Lolliguncula brevis* from the North and South Atlantic, with the two groups being identified as sister species. The divergence values observed between North and South Atlantic *Lolliguncula brevis* are of the same magnitude than the divergence values observed between other congeneric species. So, the genetic data is in perfect agreement to the proposal of Zaleski and colleagues [8] for the occurrence of distinct morphotypes within the range of *Lolliguncula brevis.* In this case, as the type locality of the species is Rio de Janeiro [23], *Lolliguncula brevis* would be the species name valid for the population from the South Atlantic. However, as proposed by Zaleski and colleagues [8], a major redescription of North and South Atlantic forms will be indispensable for the validation of *Lolliguncula brevis* from the South Atlantic, and for revalidation of *Lolliguncula brevis* from the North Atlantic, where there are at least four synonyms [1].

## Supporting Information

Table S1 **Codes, specimens, sampling localities and sequences utilized in present study.** All sequences belonging to published papers have the original references cited after the GenBank accession codes.
(DOCX)

Table S2 **Sequences utilized for the estimation of nucleotide divergence between different species of Family Loliginidae.** Sequences were obtained from Sales et al [20].
(DOCX)
