# Correction: Molecular Phylogeny of the Genus *Lolliguncula* Steenstrup, 1881 Based on Nuclear and Mitochondrial DNA Sequences Indicates Genetic Isolation of Populations from North and South Atlantic, and the Possible Presence of Further Cryptic Species

**DOI:** 10.1371/journal.pone.0102853

**Published:** 2014-07-10

**Authors:** The PLOS ONE Staff

The legends for Figures 2 and 3 are incorrect. The authors have provided corrected versions here.

**Figure 2:**
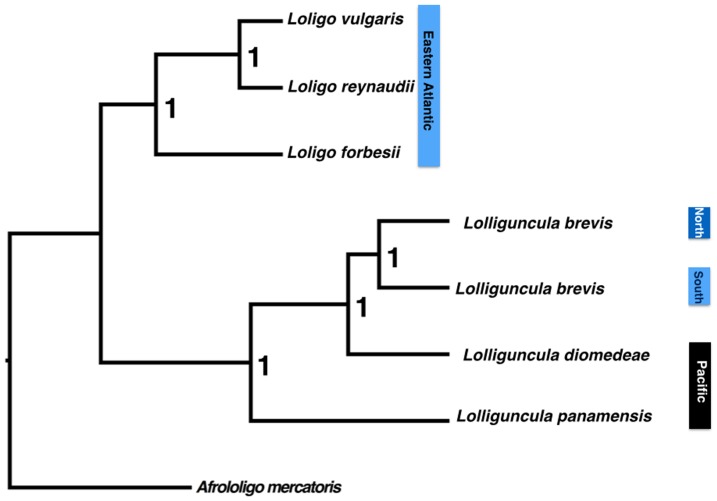
Multilocus species tree of the genus *Lolliguncula* based in two mitochondrial (16S and COI) and one nuclear genes (Rhod) obtained in the *BEAST program. Posterior probability values are shown at the nodes.

**Figure 3:**
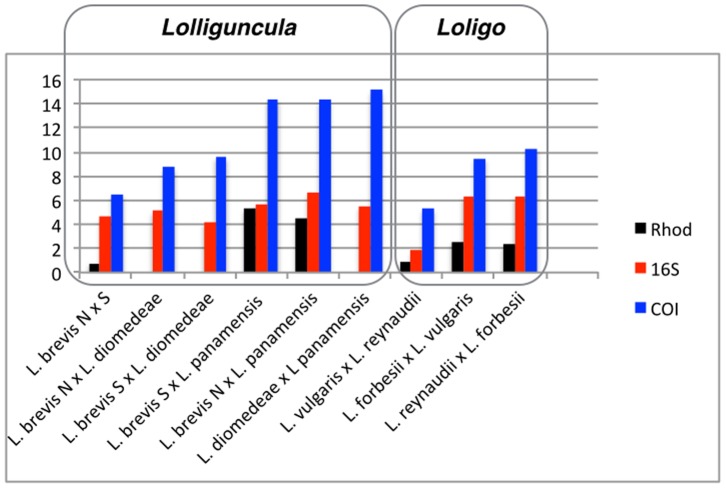
Nucleotide divergence (p) between different species of the Family Loliginidae for some pairs of congeneric species.
