# Supplementary material for: Molecular Phylogeny of the Genus Lolliguncula Steenstrup, 1881 Based on Nuclear and Mitochondrial DNA Sequences Indicates Genetic Isolation of Populations from North and South Atlantic, and the Possible Presence of Further Cryptic Species
Source: PLoS One. 2014 Feb 25;9(2):e88693. doi: 10.1371/journal.pone.0088693 (PMC3934857; doi:10.1371/journal.pone.0088693)
Supplement: Table S1 — Codes, specimens, sampling localities and sequences utilized in present study. All sequences belonging to published papers have the original references cited after the GenBank accession codes. (DOCX) [file pone.0088693.s001.docx]

Table S1: Codes, specimens, sampling localities and sequences utilized in present study. All sequences belonging to published papers had the origin references after the GenBank accession codes.

| **Code** | **Species** | **Origin/Sampling localities** | **16S** | **COI** | **Rhodopsin** |
| --- | --- | --- | --- | --- | --- |
| Amer1 | *Afrololigo mercatoris* | South Africa | AF110085[5] | AF075390[5] | EU668060[9] |
| LbrSA663 | *Lolliguncula brevis* | Pena Island near Salinas, Pará state, Brazil | KF266728 | KF266740 | KF266752 |
| LbrSA664 | *Lolliguncula brevis* | Pena Island near Salinas, Pará state, Brazil | KF266729 | KF266741 | KF266753 |
| LbrSA665 | *Lolliguncula brevis* | Pena Island near Salinas, Pará state, Brazil | KF266730 | KF266742 | KF266754 |
| LbrSA666 | *Lolliguncula brevis* | Pena Island near Salinas, Pará state, Brazil | KF273939 | KF273944 | KF273953 |
| LbrSA667 | *Lolliguncula brevis* | Pena Island near Salinas, Pará state, Brazil | KF273938 | KF273945 | KF273954 |
| LbrSA668 | *Lolliguncula brevis* | Pena Island near Salinas, Pará state, Brazil | KF273935 | KF273946 | KF273955 |
| LbrSA669 | *Lolliguncula brevis* | Pena Island near Salinas, Pará state, Brazil | KF273940 | KF273947 | KF273956 |
| LbrSA670 | *Lolliguncula brevis* | Pena Island near Salinas, Pará state, Brazil | KF273937 | KF273948 | KF273957 |
| LbrSA671 | *Lolliguncula brevis* | Pena Island near Salinas, Pará state, Brazil | KF273936 | KF273949 | KF273958 |
| LbrSA672 | *Lolliguncula brevis* | Pena Island near Salinas, Pará state, Brazil | KF273934 | KF273950 | KF273959 |
| LbrSA358 | *Lolliguncula brevis* | Baia da Traição, Paraíba state, Brazil | KF266725 | KF266737 | KF266749 |
| LbrSA359 | *Lolliguncula brevis* | Baia da Traição, Paraíba state, Brazil | KF266726 | KF266738 | KF266750 |
| LbrSA360 | *Lolliguncula brevis* | Baia da Traição, Paraíba state, Brazil | KF266727 | KF266739 | KF266751 |
| LbrSA243 | *Lolliguncula brevis* | Jequié, Bahia state, Brazil | KF266724 | KF266736 | KF266748 |
| LbrSA244 | *Lolliguncula brevis* | Jequié, Bahia state, Brazil | KF273933 | KF273943 | KF273952 |
| LbrSA818 | *Lolliguncula brevis* | Guaiabim Beach near Valença, Bahia State, Brazil | KF266731 | KF266743 | KF266755 |
| LbrSA198 | *Lolliguncula brevis* | Grauçá Beach near Caravelas, Bahia State, Brazil | KF273941 | KF273942 | KF273951 |
| LbrSA200 | *Lolliguncula brevis* | Grauçá Beach near Caravelas, Bahia State, Brazil | KF266723 | KF266735 | KF266747 |
| LbrGM1004 | *Lolliguncula brevis* | Ciudad del Carmen,Southern Gulf of Mexico | KF854126 | KF854136 | KF854145 |
| LbrGM1005 | *Lolliguncula brevis* | Ciudad del Carmen,Southern Gulf of Mexico | KF854127 | KF854137 | - |
| LbrGM1006 | *Lolliguncula brevis* | Ciudad del Carmen,Southern Gulf of Mexico | KF854128 | - | - |
| LbrGM1007 | *Lolliguncula brevis* | Ciudad del Carmen,Southern Gulf of Mexico | KF854129 | KF854138 | KF854146 |
| LbrGM1008 | *Lolliguncula brevis* | Ciudad del Carmen,Southern Gulf of Mexico | KF854130 | KF854139 | KF854147 |
| LbrGM1009 | *Lolliguncula brevis* | Ciudad del Carmen,Southern Gulf of Mexico | KF854131 | KF854140 | KF854148 |
| LbrGM1010 | *Lolliguncula brevis* | Ciudad del Carmen,Southern Gulf of Mexico | KF854132 | KF854141 | KF854149 |
| LbrGM1011 | *Lolliguncula brevis* | Ciudad del Carmen,Southern Gulf of Mexico | KF854133 | KF854142 | - |
| LbrGM1012 | *Lolliguncula brevis* | Ciudad del Carmen,Southern Gulf of Mexico | KF854134 | KF854143 | KF854150 |
| LbrGM1013 | *Lolliguncula brevis* | Ciudad del Carmen,Southern Gulf of Mexico | KF854135 | KF854144 | KF854151 |
| LbrNA1 | *Lolliguncula brevis* | Galveston, Texas, USA, Gulf of Mexico | AF110084[5] | AF074396[5] | AY616916[10] |
| Ldio1 | *Lolliguncula diomedeae* | East Tropical Pacific, México | EU735243[11] | EU735357[11] | - |
| Lpan912 | *Lolliguncula panamensis* | Bahia Las Animas, Gulf of California, México | KF266732 | KF266744 | KF266756 |
| Lpan913 | *Lolliguncula panamensis* | Bahia Las Animas, Gulf of California, México | KF266733 | KF266745 | KF266757 |
| Lpan914 | *Lolliguncula panamensis* | Bahia Las Animas, Gulf of California, México | KF266734 | KF266746 | KF266758 |
| Lrey7 | *Loligo reynaudii* | Tsirsirkana, South Africa | KF854035[12] | KF854073[12] | KF854111[12] |
| Lrey9 | *Loligo reynaudii* | Tsirsirkana, South Africa | KF854036[12] | KF854074[12] | KF854112[12] |
| Lvu681 | *Loligo vulgaris* | Lisbon, Portugal | KF854037[12] | KF854075[12] | KF854113[12] |
| Lvu684 | *Loligo vulgaris* | Lisbon, Portugal | KF854038[12] | KF854076[12] | KF854114[12] |
| Lfor41 | *Loligo forbesii* | West Coast of Scotland | KF854039[12] | KF854077[12] | KF854115[12] |
| Lfor89 | *Loligo forbesii* | West Coast of Scotland | KF854040[12] | KF854078[12] | KF854116[12] |
