# Supplementary material for: Molecular Phylogeny of the Genus Lolliguncula Steenstrup, 1881 Based on Nuclear and Mitochondrial DNA Sequences Indicates Genetic Isolation of Populations from North and South Atlantic, and the Possible Presence of Further Cryptic Species
Source: PLoS One. 2014 Feb 25;9(2):e88693. doi: 10.1371/journal.pone.0088693 (PMC3934857; doi:10.1371/journal.pone.0088693)
Supplement: Table S2 — Sequences utilized for the estimation of nucleotide divergence between different species of Family Loliginidae. Sequences were obtained from Sales et al [20]. (DOCX) [file pone.0088693.s002.docx]

Table 2S - Sequences utilized for the estimation of nucleotide divergence between different species of Family Loliginidae. Sequences were obtaind from Sales and colleagues, 2013.

| **Code** | **Species** | **Origin/Sampling localities** | **16S** | **COI** | **Rhodopsin** |
| --- | --- | --- | --- | --- | --- |
| Dple443 | *Doryteuthis* *plei* | Salinas, Pará State | KF854012 | KF854050 | KF854088 |
| Dple357 | *Doryteuthis* *plei* | Baia da Traição, Paraíba State | KF854013 | KF854051 | KF854089 |
| Dple338 | *Doryteuthis* *plei* | Iracema, Santa Catarina State | KF854014 | KF854052 | KF854090 |
| Dpea118 | *Doryteuthis* *pealei* | Bragança, Pará State | KF854015 | KF854053 | KF854091 |
| Dpea119 | *Doryteuthis* *pealei* | Bragança, Pará State | KF854016 | KF854054 | KF854092 |
| Dpea131 | *Doryteuthis* *pealei* | Bragança, Pará State | KF854017 | KF854055 | KF854093 |
| Dsur43 | *Doryteuthis surinamensis* | Cabo Norte, Amapá State | KF854018 | KF854056 | KF854094 |
| Dsur45 | *Doryteuthis surinamensis* | Cabo Norte, Amapá State | KF854019 | KF854057 | KF854095 |
| Dsur47 | *Doryteuthis surinamensis* | Cabo Norte, Amapá State | KF854020 | KF854058 | KF854096 |
| Dsan292 | *Doryteuthis* sanpaulensis | Rio Grande, Rio Grande do Sul State | KF854021 | KF854059 | KF854097 |
| Dsan293 | *Doryteuthis* sanpaulensis | Rio Grande, Rio Grande do Sul State | KF854022 | KF854060 | KF854098 |
| Dple30 | *Doryteuthis* *plei* | Gulf of Mexico | KF854023 | KF854061 | KF854099 |
| Dple36 | *Doryteuthis* *plei* | Gulf of Mexico | KF854024 | KF854062 | KF854100 |
| Dple212 | *Doryteuthis* *plei* | Gulf of Mexico | KF854025 | KF854063 | KF854101 |
| Dpea12 | *Doryteuthis* *pealei* | Rhode Island-USA | KF854026 | KF854064 | KF854102 |
| Dpea14 | *Doryteuthis* *pealei* | Rhode Island-USA | KF854027 | KF854065 | KF854103 |
| Dgah2 | *Doryteuthis* *gahi* | Falkland Islands | KF854028 | KF854066 | KF854104 |
| Dgah3 | *Doryteuthis* *gahi* | Falkland Islands | KF854029 | KF854067 | KF854105 |
| Dgah6 | *Doryteuthis* *gahi* | Falkland Islands | KF854030 | KF854068 | KF854106 |
| Dopa3 | *Doryteuthis* *opalescens* | California-USA | KF854031 | KF854069 | KF854107 |
| Dopa7 | *Doryteuthis* *opalescens* | California-USA | KF854032 | KF854070 | KF854108 |
| Lrey7 | *Loligo reynaudii* | Tsirsirkana, South Africa | KF854035 | KF854073 | KF854111 |
| Lrey9 | *Loligo reynaudii* | Tsirsirkana, South Africa | KF854036 | KF854074 | KF854112 |
| Lvu681 | *Loligo vulgaris* | Lisbon, Portugal | KF854037 | KF854075 | KF854113 |
| Lvu684 | *Loligo vulgaris* | Lisbon, Portugal | KF854038 | KF854076 | KF854114 |
| Lfor41 | *Loligo forbesi* | West Coast of Scotland | KF854039 | KF854077 | KF854115 |
| Lfor89 | *Loligo forbesi* | West Coast of Scotland | KF854040 | KF854078 | KF854116 |
| Hblk1 | *Heterololigo bleekeri* | Kingo Ito, Japan | KF854033 | KF854071 | KF854109 |
| Hblk2 | *Heterololigo bleekeri* | Kingo Ito, Japan | KF854034 | KF854072 | KF854110 |
| Uchi22 | *Uroteuthis chinensis* | Terengganu, Malasia | KF854041 | KF854079 | KF854117 |
| Usib35 | *Uroteuthis sibogae* | Terengganu, Malaysia | KF854042 | KF854080 | KF854118 |
| Usib36 | *Uroteuthis sibogae* | Terengganu, Malaysia | KF854043 | KF854081 | KF854119 |
| Uduv23 | *Uroteuthis duvauceli* | Terengganu, Malaysia | KF854044 | KF854082 | KF854120 |
| Uduv27 | *Uroteuthis duvauceli* | Terengganu, Malaysia | KF854045 | KF854083 | KF854121 |
| Uduv29 | *Uroteuthis duvauceli* | Terengganu, Malaysia | KF854046 | KF854084 | KF854122 |
| Ssep241 | *Sepioteuthis sepioidea* | Barra Grande, Bahia State, Brazil | KF854047 | KF854085 | KF854123 |
| Sles20 | *Sepioteuthis lessiniana* | Durban, South Africa | KF854048 | KF854086 | KF854124 |
| Sles21 | *Sepioteuthis lessoniana* | Durban, South Africa | KF854049 | KF854087 | KF854125 |
